# Distinguishing Antioxidant Molecules with Near-Infrared Photoluminescence of DNA-Wrapped Single-Walled Carbon Nanotubes

**DOI:** 10.1021/acsomega.2c02038

**Published:** 2022-08-08

**Authors:** Nay San Lin, Masaki Kitamura, Makoto Saito, Kota Hirayama, Yuki Ide, Kazuo Umemura

**Affiliations:** Department of Physics, Tokyo University of Science, 1-3 Kagurazaka, Shinjuku, Tokyo 1628601, Japan

## Abstract

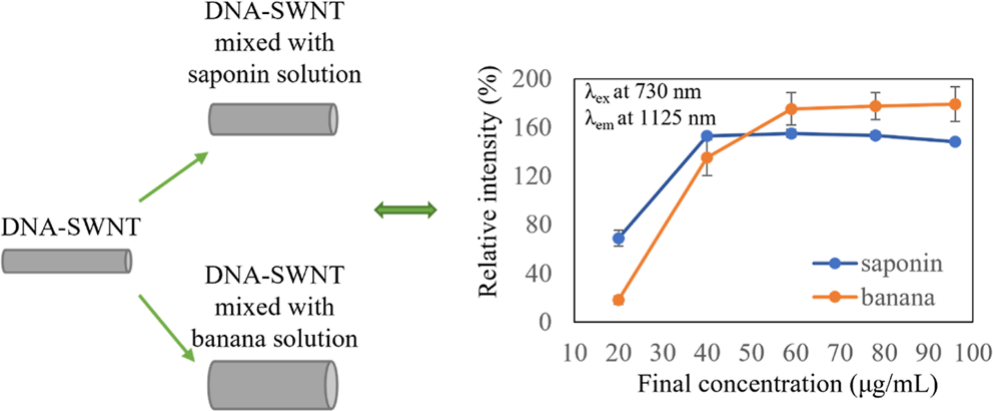

In this study, two biomolecule solutions were distinguished using the capacity difference in the near-infrared photoluminescence (PL) of single-walled carbon nanotubes (SWNTs). Biosensing techniques using sensitive responses of SWNTs have been intensively studied. When a small amount of an oxidant or reductant solution was injected into the SWNT suspensions, the PL intensity of the SWNTs is significantly changed. However, distinguishing between different molecules remains challenging. In this study, we comparably injected saponin and banana solutions, which are known antioxidant chemicals, into an SWNT suspension. The SWNTs were solubilized by wrapping them with DNA molecules. The results show that 69.1 and 155.2% increases of PL intensities of SWNTs were observed after injection of 20 and 59 μg/mL saponin solutions, respectively. Subsequently, the increase in PL was saturated. With the banana solution, 18.1 and 175.4% increases in PL intensities were observed with 20 and 59 μg/mL banana solutions, respectively. Based on these results, the two antioxidant molecules could be distinguished based on the different PL responses of the SWNTs. In addition, the much higher saturated PL intensities observed with the banana solution suggests that the banana solution increased the capacity of the PL increase for the same SWNT suspension. These results provide helpful information for establishing biosensing applications of SWNTs, particularly for distinguishing chemicals.

## Introduction

Single-walled carbon nanotubes (SWNTs) are graphene sheets rolled into cylinders and arranged with hexagonal carbon rings by means of chiral indices *n* and *m*.^1^ The *n* and *m* values, presented in the form (*n*,*m*) denoted as chirality, provide information about the diameters of SWNTs and chiral angles of the hexagons of carbon atoms when they are rolled into cylindrical tubes.^2^ Chirality also provides information about the types of SWNTs; depending on the n and m indices, SWNTs can be categorized as chiral or achiral and can be further classified as zigzag and armchair.^3^ Moreover, chiral indices can differentiate metallic from semiconductor SWNTs.^4^

SWNTs exhibit excellent electrical properties. They have highly stable characteristics,^5^ and their highest advantage is that their band gaps can be regulated.^6^ Sankar and Kumar reported that in semiconducting SWNTs, the electron band gap is inversely proportional to the diameter. Moreover, they have high strength owing to their superior atomic structures as they are strongly composed of carbon bonds which are stronger than those of diamonds.^7^

Furthermore, the optical properties of SWNTs have been widely studied. SWNTs has been reported to exhibit optical properties such as absorption and fluorescence, and Blancon et al. observed non-resonant absorption in SWNTs, using spatial modulation spectroscopy.^8^ Lee et al. reported that the fluorescent efficiency is insignificant in SWNTs, and it was enhanced by wrapping SWNTs with DNA and using reducing agents and also reported that SWNTs have intrinsic characteristics, which make them bright emitters.^9^ Wei et al. remarked that to analyze the structures of SWNTs, such as family, diameter, and chiral angle, photoluminescence (PL) quantum yield is necessary and observed that PL intensities depended on the concentration of SWNTs.^10^

The chemical interactions between SWNTs and molecules have attracted significant research attention. Matsuura et al. studied the selectivity of water-soluble proteins by SWNTs by dispersion, and they found that egg white lysozyme and bovine serum albumin could disperse SWNTs, whereas papain and pepsin could not.^11^ The SWNTs’ selectivity of nitrogen oxide molecules,^12^ sulfur dioxide molecules,^13^ porphyrins,^14^ peanut allergy-causing protein ara h1,^15^ and pyrene molecules,^16^ as well as their interactions have been reported. Card et al. pointed out that SWNTs generally have selective sensitivities to single molecules of analytes.^17^

Saponins are generally derived from plants and aquatic organisms and are known as glycosides composed of triterpenes and steroids.^18,19^ Saponins are useful owing to their enormous beneficial effects on animal health. To date, saponins have shown their ability to digest cholesterol and their anticancer, antioxidant, and antibacterial properties.^20^ In contrast, banana is a tropical fruit that is amply grown in Asia and has various capacities that bring noble health benefits. Moreover, bananas provide antioxidant potencies not only in the pulp but also in the peel, which can have beneficial applications.^21^ Banana is composed of antioxidant-rich components, such as gallocatechin,^22−24^ dopamine,^22^ phenolic acids, caffeic acids, and hydroxybenzoic acids.^25^

The antioxidant potencies of saponin and banana solutions have been analyzed using traditional methods by various research groups.^20,21,25−32^ However, the utilization of the optical responses of DNA-wrapped SWNTs (DNA-SWNTs) in the near-infrared (NIR) region has promised a modern technique for analyzing and distinguishing antioxidant biomolecules. Hamano et al. studied the antioxidant ability of catechin using NIR absorbance (NIR-ABS) values of DNA-SWNTs,^33^ whereas Matsukawa and Umemura analyzed it by means of NIR photoluminescence (NIR-PL) intensities of DNA-SWNTs,^34^ and Yamazaki and Umemura analyzed the antioxidant potencies of epigallocatechin gallate and tannic acid using NIR-PL and NIR-ABS, and the NIR-PL results showed higher antioxidant capacities.^35^ NIR-PL spectroscopy was employed to assess the optical responses of DNA-SWNTs to an oxidant (KMnO_4_) and antioxidant biomolecules (saponin and banana biomolecules). The differences in the PL responses of SWNTs, depending on their chemical interactions with different molecules, such as DNA and biomolecules, were successfully investigated. Moreover, having different PL intensity responses upon injecting two different antioxidant biomolecules, DNA-SWNTs successfully recognized these biomolecules by showing different PL response capacities. Therefore, multiple biomolecules were successfully investigated using a single biosensing tool. NIR-ABS measurements were conducted to confirm the PL capacity of DNA-SWNTs in distinguishing between the two biomolecules. Using atomic force microscopy (AFM), different PL intensities were confirmed depending on the diameter of the DNA-wrapped SWNTs after mixing with the two biomolecules. In summary, this study provides crucial information in the field of biosensing, specifically for distinguishing chemicals.

## Results and Discussion

DNA-SWNTs were used to distinguish between biomolecules in the NIR region, using PL measurements. In the NIR-PL measurements, the intensities of the emitted light spectra were determined with the aid of mapping. The various colors in the PL mapping represent only the intensities in the NIR region. The bright spots in the PL maps represent the intensities representing certain chiral indices. Semiconducting SWNTs were prominent in the excitation wavelength range of 500–800 nm and the emission wavelength range of 900–1300 nm, and the red area in top left corner of the PL map represents the Rayleigh scattering with a high intensity (Figures S5 and S6). In this study, (7,5) and (7,6) chiralities were prominent at an excitation wavelength of 655 nm, whereas (10,2) and (9,4) were prominent at 730 nm. For numerical analysis, the focus was on the (7,5), (7,6), and (9,4) chiralities, whereas the (9,4) chirality was focused on because it showed the highest recovery to the initial state.

**Figure 1:**
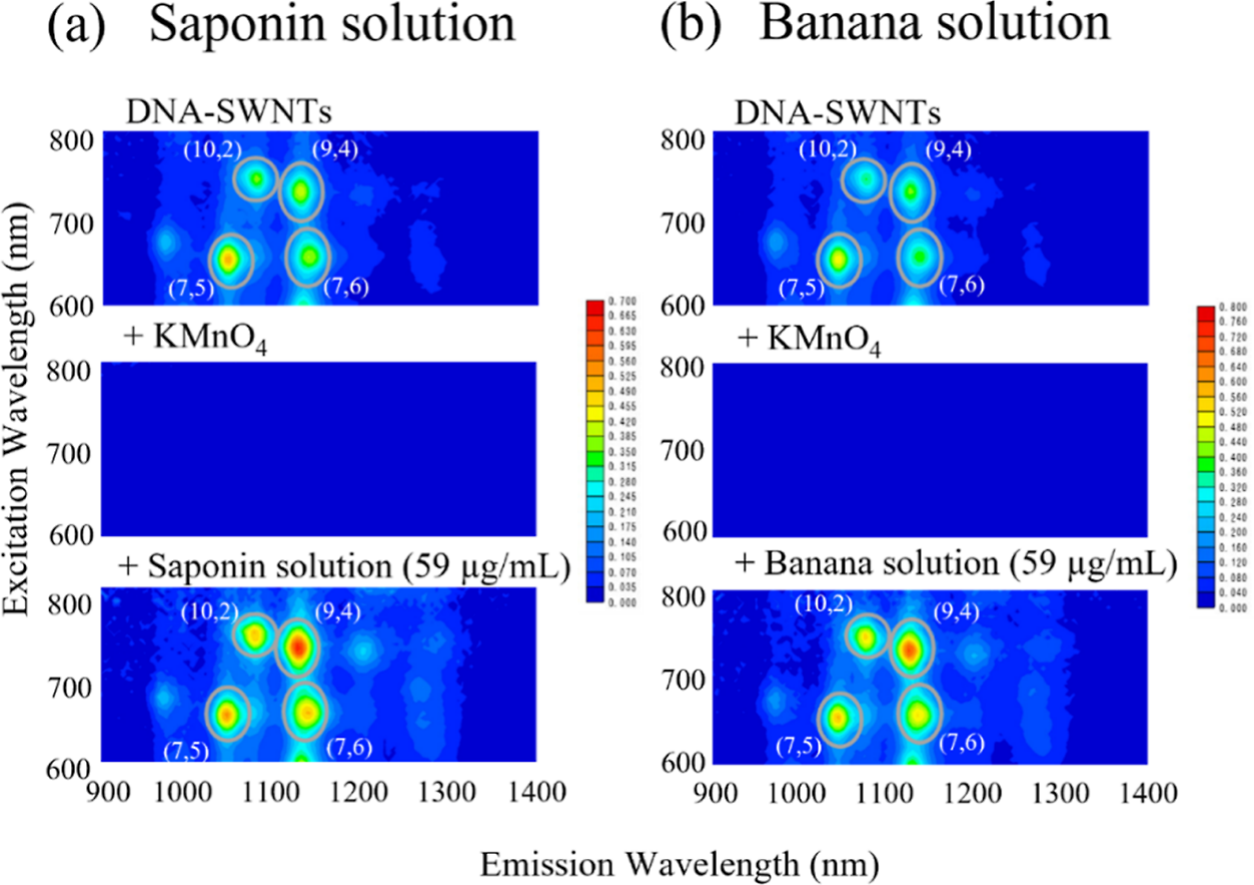
Mapping of the NIR-PL spectra of the DNA-SWNTs with and without an oxidant and reductant. From top to bottom: DNA-SWNTs (initial state), injected KMnO_4_ (state of oxidation), injected biomolecules after KMnO_4_ treatment (state of reduction). In the state of reduction, the biomolecules utilized were (a) saponin and (b) banana solutions.

Figure 1 shows the PL maps of the spectra of DNA-SWNTs with and without the injection of KMnO_4_ and biomolecule solutions. The uppermost PL map represents the initial state of the DNA-SWNTs before injection of a solution of biomolecules. When DNA-SWNTs were oxidized with KMnO_4_ (final concentration of 0.5 μM), the PL intensities were quenched completely, resulting in no remarkable PL intensities. Dukovic et al. elucidated that the merits of SWNTs, such as electrical conductivity and photoconductivity, are attributed to the occurrence of highly active optical transitions in excited states, and consequently, they become sensitive to quenching.^36^

When saponin or banana solutions were injected as antioxidant biomolecules into the DNA-SWNTs, the PL intensities recovered. Oxidation can be defined as a chemical process of electron loss, whereas reduction can be defined as an electron-gaining process during a chemical reaction.^37^ Antioxidants have properties that are against oxidation, and they have a tendency to donate electrons and generally donate electrons to free radicals^38−40^ which are atoms or molecules with unpaired valence electrons.^41^ They also exhibit the properties of reducing agents or reductants.^42^ Because antioxidants have properties that are against oxidation in many ways,^43−45^ the quenched PL intensities were recovered against the state of oxidation, after the injection of antioxidant biomolecules.

In this figure, among all final concentrations, the final concentration of 59 μg/mL was chosen to illustrate the state of reduction because it fully or closely contributed to the saturation of PL intensities of the (9,4) chirality in both cases; additionally, it was attributed to the saturation of the (7,5) chirality in the banana solution case. In the initial state, the (7,5) chirality was the most remarkable; however, in the state of reduction, the (9,4) chirality became the most remarkable at a final concentration of 59 μg/mL. Tanaka et al. investigated the chirality-dependent redox potentials of SWNTs.^46^ According to the numerical values of their experiments, the oxidation potential of (7,5) SWNTs was the highest and that of (9,4) SWNTs was the lowest.^46^ Hence, (9,4) SWNTs may undergo fewer oxidation reactions with oxidants, whereas (7,5) SWNTs were oxidized with KMnO_4_ the most. Therefore, the least PL intensity recoveries from the PL quenching in the oxidation state are expected for (7,5) SWNTs. In contrast, most PL intensity recoveries are expected for (9,4) SWNTs. Additionally, it was observed that the electrochemical band gaps of the (7,5) chirality SWNTs were larger than those of the (9,4) chirality SWNTs.^46^ Therefore, it is speculated that the (9,4) chirality SWNTs exhibit the highest PL intensity in the state of reduction.

**Figure 2:**
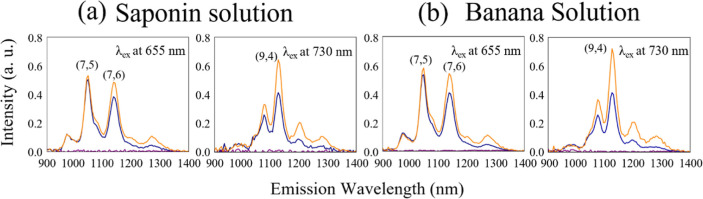
NIR-PL Spectra of the DNA-SWNTs with and without an oxidant and reductant. Navy blue: DNA-SWNTs (initial state). Purple: injected with KMnO_4_ (state of oxidation). Orange: injected with (a) saponin or (b) banana solutions after KMnO_4_ treatment (state of reduction).

Figure 2 shows the NIR-PL spectra of DNA-SWNTs with and without the injection of KMnO_4_ and solutions of antioxidant biomolecules. In the initial state (navy blue line) of the saponin solution case, the PL intensities of the (7,5), (7,6), and (9,4) chirality SWNTs are 0.505 ± 0.008, 0.384 ± 0.006, and 0.414 ± 0.009, respectively (Table S1). Different chiralities produce different PL intensities. Among all these three chirality SWNTs, (7,5) SWNTs have the smallest diameters.^47,48^ Since light absorption and emission of smaller-diameter SWNTs are superior to those of larger ones,^48,49^ and it can be said that PL intensity has an inverse relationship with diameters of SWNTs,^48^ it is clear that (7,5) SWNTs showed the highest intensity in the initial state.

In the KMnO_4_-oxidized state (purple line), no significant peaks were observed because this is the state in which PL quenching occurs, as shown in the PL maps. When a saponin solution with a final concentration of 59 μg/mL (orange line) was injected, the PL intensities were recovered from the state of oxidation, with numerical values of 0.532 ± 0.013, 0.486 ± 0.009, and 0.643 ± 0.018 for the (7,5), (7,6), and (9,4) chirality SWNTs, respectively.

For the banana solution, the PL intensities of the initial state (navy blue line) were 0.533 ± 0.016, 0.407 ± 0.023, and 0.415 ± 0.046, respectively (Table S2). No peaks were prominent in the oxidation state (purple line) or in the state of reduction (orange line); the PL intensities were 0.581 ± 0.013, 0.540 ± 0.020, and 0.721 ± 0.033, for the (7,5), (7,6), and (9,4) chirality SWNTs, respectively.

Focusing on the initial states, the (7,5) SWNTs exhibited the highest PL intensities in both biomolecule solution cases. When solutions of biomolecules with a final concentration of 59 μg/mL were injected, the (9,4) SWNTs exhibited the highest recoveries in PL intensities, as shown in the PL maps. Mistry et al. wrapped large-diameter SWNTs with two different kinds of polymers: PFO-BPy and PFO-A. According to their observations, the former polymer did not show any selectivity toward these large-diameter SWNTs, whereas the latter showed interactions with certain chiralities.^50^ Therefore, it is also speculated in this study that the selectivity of SWNTs may be one of the factors responsible for the sudden PL intensity increases in the case of the (9,4) chirality when solutions of biomolecules were injected. Because DNA and the other two biomolecules are different types of molecules, the selectivity of different SWNT chiralities by these molecules at the initial state may be different from that of the reduced state.

**Figure 3:**
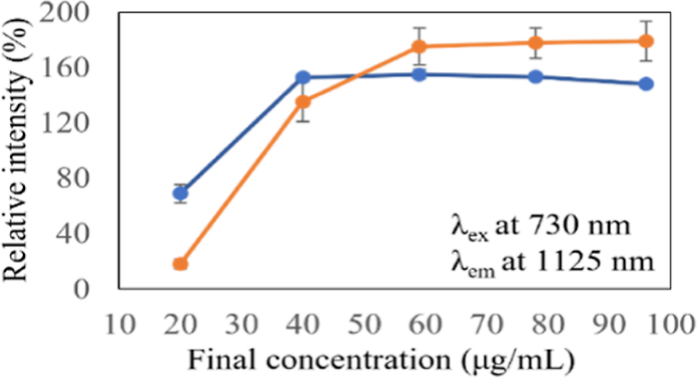
Comparison of relative PL intensities of DNA-SWNTs, when KMnO_4_ and saponin (blue line) or banana (orange line) solutions were added. The relative intensity vs final concentration graph is made by using results of (9,4) chirality peaks of DNA-SWNTs at an excitation wavelength of 730 nm and an emission wavelength of 1125 nm. Error bars are illustrated by using standard error values.

Figure 3 shows the graph of relative intensity vs the final concentration. Herein, the relative intensity is defined as the ratio of the PL intensity of DNA-SWNTs to that of DNA-SWNTs after the injection of KMnO_4_ and the antioxidant biomolecule solutions. In other words, it is the ratio of the PL intensity achieved in the initial state to the PL intensity achieved in the state of reduction. The graph reveals two main points: how PL intensities vary with the final concentrations of solutions of biomolecules and how biomolecules are distinguished. This graph focuses on the PL intensities of the (9,4) chirality of DNA-SWNTs when different concentrations of biomolecule solutions were injected. The PL intensities of the (7,5) and (7,6) chiralities for different concentrations of biomolecule solutions are presented in the Supporting Information (Tables S1 and S2). In the current graph, a final concentration of 20 μg/mL revealed weak recovery rates in both biomolecule cases, and the recovery percentage of the spectra of DNA-SWNTs when the banana solution was injected was lower than that of the saponin solution. If the PL intensity of the initial state is taken to be 100%, the recovery percentages were 69.1 and 18.1% for the saponin and banana solutions, respectively. At 40 μg/mL, the recovery rates went up drastically to 153.2 and 135.4%, respectively; however, the recovery of DNA-SWNTs was still weaker in the case of the banana solution. Starting from 59 μg/mL, the recovery rates of the PL intensities became saturated in both cases; however, the PL intensities increased when the banana solution was injected, even though they were weaker at lower concentrations. The recovery percentages were 155.2% in the saponin solution case and 175.4% in the banana solution case. In the case of the banana solution, the PL intensities continued to saturate up to 96 μg/mL, with 179.2% recovery rates; however, the recovery rates of the PL intensities dropped to 148.2% in the case of saponin solution. This may be due to the dilution of SWNTs (a decrease in the final concentration of SWNTs) when more saponin solution was injected. The results show that the recovery percentages of PL intensities were slightly higher in the case of the banana solution than those in the case of the saponin solution, even though the same suspension of SWNTs and final concentrations of solutions of biomolecules were used. This reveals that the banana solution expanded the optical responses of DNA-SWNTs. Therefore, DNA-SWNTs are useful biosensing tools for distinguishing biomolecules, based on NIR-PL intensities. Card et al. pointed out that SWNTs show heterogeneity in optical characteristics depending on the analytes that they react with.^17^ Different analytes produce different variations in optical responses. This may explain why different optical responses of DNA-SWNTs were achieved when saponin and banana solutions were injected.

NIR-ABS measurements were conducted to validate the antioxidant properties of the two molecules distinguished by the NIR-PL measurements (Figures S7 and S8). The observed chiralities were speculated to be (7,5), (8,4)/(9,4)/(7,6), (8,6)/(12,1)/(11,3), and (10,5)/(8,7).^33,47,51^ After oxidation with KMnO_4_, the antioxidant abilities of the two biomolecule solutions were noticeably different. The recovery percentages of the saponin solution were lower than those in the initial state for all chiralities. In contrast, the banana solution exhibited higher recovery percentages compared with the initial state for (10,5)/(8,7) chirality. Hence, it was observed that the saponin solution did not fully recover the NIR-ABS spectra of all SWNTs oxidized by KMnO_4_, whereas the banana solution enhanced the absorbance peak of a certain chirality. Therefore, these two biomolecules can be distinguished.

The antioxidant abilities of the two biomolecules were investigated without adding KMnO_4_, by employing NIR-PL spectroscopy (Figures S9–S11). Without oxidation, the PL enhancements were higher than those of the previous redox reactions. When the two biomolecule solutions were compared, the banana solution enhanced the PL spectra of all (7,5), (7,6), and (9,4) SWNTs more than the saponin solution, even without the oxidation with KMnO_4_. Even without the oxidation process, the (9,4) SWNTs showed the highest PL intensities after reduction with antioxidant biomolecules, whereas the (7,5) SWNTs showed the highest PL intensities in the initial state (Figures S9–S11, Tables S3 and S4). Even though largely strong oxidant KMnO_4_ was not used, we speculate that it is possible that the DNA-SWNT suspension would be oxidized by the oxygen in the air through the passage of time since the experiments were carried out in open conditions. Since (9,4) SWNTs have the lowest oxidation potential,^46^ it is clear that they showed the highest recovery from the oxidation at the state of reduction. However, it is speculated that the oxidation was much weaker than that with KMnO_4_, and the recovery rates were higher in this case. According to NIR-ABS, measurements showed that the banana solution enhanced the NIR-ABS spectra of all chiral SWNTs without the oxidation process with KMnO_4_, showing its highest recovery percentage of 109.0% for the (10,5)/(8,7) chirality (Figure S8 and Table S5). In contrast, saponin solution did not enhance the NIR-ABS spectra of all chiralities, except (10,5)/(8,7) chirality, and the highest recovery percentages were obtained as only 102.2% for (10,5)/(8,7) chirality (Figure S8 and Table S5). Hence, these two biomolecules were successfully distinguished in various aspects.

For the absorbance measurements, the spectra in the visible region (600–800 nm), which was used for the excitation wavelength range in NIR-PL measurements, was determined. If the 800–1350 nm range represents E_11_ optical transitions, the 600–800 nm wavelength range is assumed as the representation of E_22_ optical transitions. Peak 1 and Peak 2 were seen at 654 and 733 nm. 654 nm is speculated as the excitation wavelength for (7,5) and (7,6) chiralities, and 733 nm is for (9,4) chirality.^47,52^ Hence, these results were consistent with those of NIR-PL measurements. For Peak 1 and Peak 2, absorbance enhancements were superior in the case of banana solution than saponin solution (Figures S7 and S8, Table S6), even though the difference was slightly less prominent. We distinguished the two molecules from another aspect.

**Figure 4:**
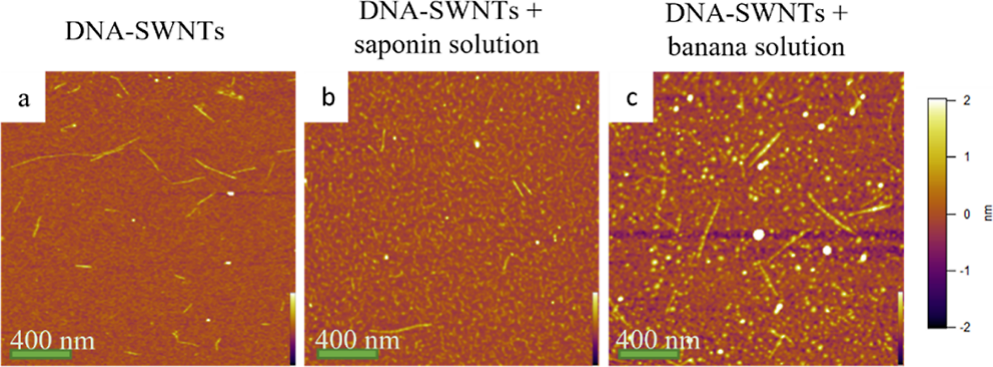
AFM images of (a) DNA-SWNTs and (b) DNA-SWNTs mixed with saponin solution and (c) DNA-SWNTs mixed with banana solution. Each solution was diluted 25 times with Tris-HCl buffer and analyzed in AFM-air conditions after dropping onto AP-treated mica substrates.

AFM measurements were performed in air to perform structural analysis of the DNA-SWNTs. DNA-wrapped SWNTs are clearly visible in Figure 4a. A total of 100 heights were randomly selected using 20 SWNTs. The average diameter was 1.03 ± 0.41 nm. Generally, the diameters of individual HiPCO SWNTs range from 0.7 to 1.4 nm,^53^ and in this study, the distribution of diameters between 0.7 and 1.4 nm was 63%. In Figure 4b,c, DNA-SWNTs were clearly observed, even after mixing with saponin or banana solutions. The average diameters of DNA-SWNTs mixed with saponin or banana solutions were 1.32 ± 0.48 and 1.74 ± 1.71 nm, respectively. The distributions of the individual diameters ranging from 0.7 to 1.4 nm were 43 and 49%, respectively. The heights of 100 gold colloids were measured to correct the diameters owing to tip convolution effects. The average height was measured as 3.57 ± 0.76 nm. After correction with the heights of the gold colloids, the average diameter of the DNA-SWNTs was calculated as 1.44 ± 0.58 nm.

The distribution of diameters between 0.7 and 1.4 nm was calculated as 55%. Therefore, it can be speculated that the SWNTs were abundantly dispersed individually, and some of them were dispersed as small bundles. The average diameters of DNA-SWNTs mixed with saponin or banana solutions were calculated as 1.85 ± 0.67 and 2.44 ± 2.40 nm, and the distributions of the individual diameters ranging from 0.7 to 1.4 nm were 20 and 36%, respectively.

**Figure 5:**
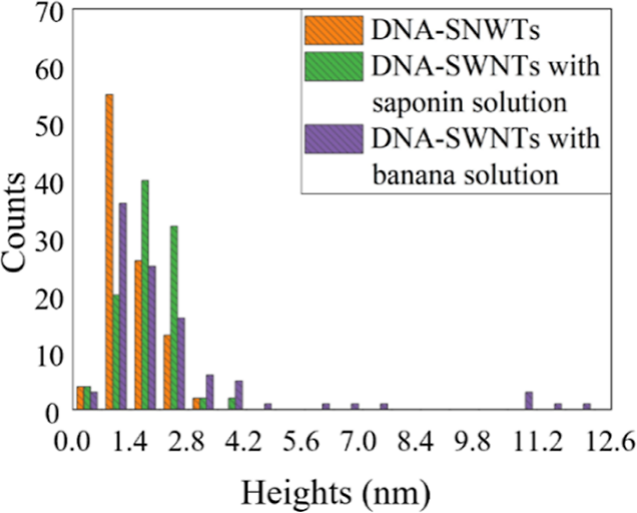
Height distribution histograms for DNA-SWNTs and those mixed with saponin and banana solutions. Histograms were illustrated using the data of the heights corrected with those of gold colloids. The bin size was chosen as 0.7 nm.

Figure 5 shows the height distributions of DNA-SWNTs and those mixed with saponin or banana solutions after correcting the diameters with those of the gold colloids. The bin size was chosen as 0.7 nm. The diameters of DNA-SWNTs were distributed in the smallest values in the histograms, whereas the diameters of DNA-SWNTs mixed with saponin or banana solutions were distributed to the right, indicating that the diameter increased. It is speculated that in the banana solution case, very large diameters (up to 12.6 nm which is nine times larger than a 1.4 nm individual SWNT) were seen, and the average diameter was also relatively large.

The diameters observed in the case of the banana solution were much larger than those in the saponin solution case, even though the percentage of individual SWNTs was slightly higher in the former case. In the saponin solution, the diameters were relatively small (up to only 4.2 nm). Bananas contain binding proteins, such as lectin.^54^ This increase in the diameter seems to be the result of protein adsorption onto the DNA-SWNTs.^55^ If proteins and SWNTs are bound by DNA owing to the electron coupling effect, the fluorescence intensity will increase.^56^ This may be the reason why the absorbance and PL enhancements of DNA-SWNTs were higher when the DNA-SWNTs were mixed with the banana solution because it was speculated that the absorbance and PL intensity would be increased remarkably when banana-binding proteins were adhered to the DNA-SWNTs. In summary, the AFM results in this study are consistent with the PL results. The histograms of the height distributions of the pure DNA-SWNTs, DNA-SWNTs mixed with saponin or banana solutions, and gold colloids are shown in Figure S13.

## Conclusions

In this study, two antioxidant biomolecules, saponin and banana, were successfully distinguished using the PL enhancement of DNA-SWNTs. Compared to the saponin solution, a saturated amount of banana solution can exhibit higher PL enhancement of DNA-SWNTs, with or without oxidation with KMnO_4_. NIR-ABS measurements supported the results of the NIR-PL measurements, even though the NIR-ABS recovery rates were lower. In the NIR-ABS measurements with the KMnO_4_ oxidation, the banana solution revealed absorbance increases in (10,5)/(8,7), whereas the saponin solution did not reveal in all SWNTs. In the absence of KMnO_4_, the banana solution showed absorbance enhancement of all SWNTs, whereas the saponin solution did not enhance the NIR-ABS spectra of all SWNTs, except (10,5)/(8,7), and showed weaker absorbance enhancements in all chiral SWNTs compared to the banana solution. Hence, the absorbance measurements successfully distinguished the two biomolecules, same as the NIR-PL measurements. Even though our research was mainly focused on the study of optical properties, it gives some insights into electrochemical properties of SWNTs. The diameter increases of the DNA-SWNTs were relatively higher when they were mixed with the banana solution than when they were mixed with the saponin solution. Hence, this could provide a useful correlation between the PL enhancement and the diameter increase of DNA-SWNTs measured by AFM. In summary, the two biomolecules were successfully distinguished in various aspects.

## Methods

In this study, the SWNTs produced by the HiPCO method were purchased from NoPo Nanotechnologies India Private Limited (India), and double stranded DNA (deoxyribonucleic acid sodium salt from salmon testes, no. D1626-250 MG), saponin (84510-100G), and banana powder (B4032-500G) were purchased from Sigma-Aldrich Co. LLC (St. Louis, MO, USA).

First, DNA was dissolved in 10 mM tris(hydroxymethyl)-aminomethane (Tris-HCl buffer solution, pH 8.0) to prepare a 1.0 mg/mL DNA solution. Dispersion was performed in a bath-type ultrasonicator (80 W) for 180 min on ice to ensure uniformity. The solution was shaken gently in an ice bath for 180 min.

To prepare DNA-SWNTs, 0.69 mg of SWNTs was dissolved in 1.38 mL of the prepared DNA solution, so that the final concentration of the solution was 0.5 mg/mL. The solution was sonicated using a probe-type sonicator (VCX130; Sonics & Materials, Inc. Newtown, CT, USA) in an ice–water bath for 90 min at 2 W. Subsequently, the solution was centrifuged for 3 h at 15,000 rpm (21,500*g*) at 8 °C. Finally, 70% of the supernatant was stored for further use after removing insoluble residual SWNTs.

Saponin was stored at room temperature (23 °C), and banana powder was stored at 3.5 °C initially. 2.55 mg of saponin was mixed with 1.275 mL of pure water, and the solution was denoted as saponin solution (concentration of 2 mg/mL) and then stored in a refrigerator for further use. Moreover, 2.33 mg of banana powder was dissolved in 1.165 mL of pure water, and the solution was named banana solution (concentration of 2 mg/mL) and then stored in the refrigerator. Saponin and banana solutions were stored at 3.5 °C.

To distinguish between the antioxidant biomolecules, PL measurements were performed using DNA-SWNTs. The PL spectra of the samples were measured using a PL spectrometer (NIR System; Shimadzu Co., Ltd., Kyoto, Japan). To determine the concentration of DNA-SWNTs, absorbance was measured using UV–vis spectrophotometry (V-630, JASCO Corp., Hachioji City, Tokyo, Japan), and the concentration at which the absorbance value was 0.1 at 808 nm was used in the PL measurements. First, the PL excitation wavelength range was chosen from 400 to 1000 nm, and the emission wavelength range was chosen from 850 to 1600 nm. For further experiments, the excitation and emission wavelength ranges were 600–800 and 900–1400 nm, respectively. PL spectroscopy is an optical spectroscopy technique in which emission wavelengths are measured in the near-infrared (NIR) region. The colors indicated in the PL maps, such as blue, green, yellow, and red, are only a visual representation of their PL intensities.

To measure the NIR-PL values, 464 μL of Tris-HCl buffer and 26 μL of the prepared DNA-SWNT solution were mixed in a cuvette, and the spectra were recorded initially. The solution was oxidized by adding 5 μL KMnO_4_ (final concentration of 0.5 μM), and after 10 min of incubation, the spectra were recorded. The prepared solution was then treated with 5 μL of saponin solution (final concentration of 20 μg/mL) or banana solution (final concentration of 20 μg/mL), and the spectra were recorded after 10 min of incubation at room temperature (23 °C). The final concentrations were varied by adding 5 μL each time, the resulting concentrations were 40, 59, 78, and 96 μg/mL, and the incubation period each time was 10 min. The experiment was conducted three times, and the results were presented as mean ± standard deviation. The experiment was repeated without the KMnO_4_ oxidation process by adding 59 μg/mL solutions of biomolecules to 490 μL of the DNA-SWNTs and Tris-HCl buffer mixture. The experiment was conducted three times, and the results were presented as mean ± standard deviation.

NIR-ABS measurements were performed using the SolidSpec-3700DUV (Shimadzu Co. Kyoto, Japan) and 980 μL of the mixture solution containing DNA-SWNT solution with Tris-HCl buffer. The concentration for which the absorbance of SWNTs is 808 nm is 0.1. The wavelength range was chosen from 800 to 1350 nm. The solution was oxidized with 10 μL KMnO_4_ (final concentration of 0.5 μM) and reduced with 30 μL of the solutions of the biomolecules (final concentration of 59 μg/mL), and the spectra were recorded at each state. The experiment was repeated without the oxidation process, by choosing the final concentration of the biomolecule solution as 59 μg/mL.

For the absorption spectra in the visible region (600–800 nm) with or without the oxidation process with KMnO_4_, experiments were carried out using UV–vis spectrophotometry (V-630, JASCO Corp., Hachioji City, Tokyo, Japan). The concentrations for DNA-SWNT, KMnO_4_, saponin, and banana solutions were the same as those used in NIR-ABS measurements.

An atomic force microscope (MFP-3D microscope, Asylum Research, Santa Barbara, CA, USA) was employed for the structural analysis of the DNA-SWNTs. Experiments were conducted in the AC-AFM mode in air with the application of a silicon cantilever PPP-NCSTR-W (NANOSENSORS, Nanoworld AG, Neuchatel, Switzerland). Mica substrates were treated with 0.01% 3-aminopropyl triethoxysilane (Shin-Etsu Chemical Co., Ltd. Tokyo, Japan) for use in the AFM measurements. The DNA-SWNT solution was diluted 25 times with Tris-HCl buffer. Then, 10 μL of the prepared sample was dropped onto the center of the mica substrates. After incubating for 10 min at room temperature, the sample was washed twice with 1 mL of pure water and dried at room temperature for one day. Finally, structural analysis of the prepared sample was performed using AFM. Twenty DNA-SWNTs were chosen randomly, and 100 heights were selected.

For the structural analysis of DNA-SWNTs mixed with saponin or banana solutions, 464 μL of Tris-HCl buffer, 26 μL of DNA-SWNT solution, 15 μL of saponin solution (saturated concentration in the PL measurement), or 25 μL of banana solution (saturated concentration in the PL measurement) were mixed together in a container in each experiment. The solution (100 μL) was mixed with 29 μL of Tris-HCl buffer again in the case of saponin solution or 26 μL of Tris-HCl buffer in the case of banana solution, so that the dilution of DNA-SWNTs was 25 times in each experiment. The subsequent processes were the same as those used for the measurement of DNA-SWNTs without the saponin solution.

In the structural analysis of saponin or banana solutions only, 519 μL of water and 15 μL of saponin solution or 515 μL of water and 25 μL of banana solution were mixed in each case. The solution (10 μL) was dropped onto the center of the mica substrates, and the same processes were followed.

Focusing on the tip convolution effects, AFM measurements were carried out by diluting 5 nm gold colloids (EM. GC5, BBI Solutions, the UK, mean diameter: 4.6–6 nm, coefficient of variation: ≤15%) 100 times with water, and 10 μL of the solution was dropped onto the center of mica substrates, and the remaining steps were the same as those with earlier experiments.

## Supporting Information Available

PL graphs of the optical responses of DNA-SWNTs after injecting KMnO_4_ and saponin solution; PL graphs of optical responses of DNA-SWNTs after injecting KMnO_4_ and banana solution; representation of chiralities; peaks of prominent chiralities; NIR-ABS spectra of DNA-SWNTs after injecting solutions of biomolecules with or without KMnO_4_; NIR-PL mapping images of DNA-SWNTs with the injection of solution of biomolecules; NIR-PL spectra of DNA-SWNTs with the injection of solution of biomolecules; percentage increases in absorbance of (7,5), (7,6), and (9,4) SWNTs in the state of reduction; AFM images of saponin, banana, and colloids; histograms of DNA-SWNTs with saponin or banana solutions; PL intensities and emission wavelengths of DNA-SWNTs with or without KMnO_4_; Absorbances and wavelengths of DNA-SWNTs with or without KMnO_4_ at the 800–1350 and 600–800 nm wavelength range (PDF)

## Abbreviations

AFM: atomic force microscopy
AP-mica: mica treated with 3-aminopropyltriethoxysilane
DNA: deoxyribonucleic acid
DNA-SWNTs: DNA-wrapped single-walled carbon nanotubes
HiPCO: high-pressure carbon monoxide
PL: photoluminescence
NIR: near-infrared
NIR-ABS: NIR absorbance
NIR-PL: NIR photoluminescence
KMnO_4_: potassium permanganate
PL: photoluminescence
SWNT: single-walled carbon nanotube
UV–vis: ultraviolet–visible
