# Supplementary material for: Distinguishing Antioxidant Molecules with Near-Infrared Photoluminescence of DNA-Wrapped Single-Walled Carbon Nanotubes
Source: ACS Omega. 2022 Aug 8;7(33):28896–903. doi: 10.1021/acsomega.2c02038 (PMC9404167; doi:10.1021/acsomega.2c02038)
Supplement: Supplementary file 1 — ao2c02038_si_001.pdf [file ao2c02038_si_001.pdf]

Supporting Information

Distinguishing Antioxidant Molecules with Near-infrared Photoluminescence of DNA-wrapped Single Walled Carbon Nanotubes

*Nay San Lin, Masaki Kitamura, Makoto Saito, Kota Hirayama, Yuki Ide, Kazuo Umemura**

Department of Physics, Tokyo University of Science,

1-3 Kagurazaka, Shinjuku, Tokyo 162-8601, Japan

*meicun2006@163.com

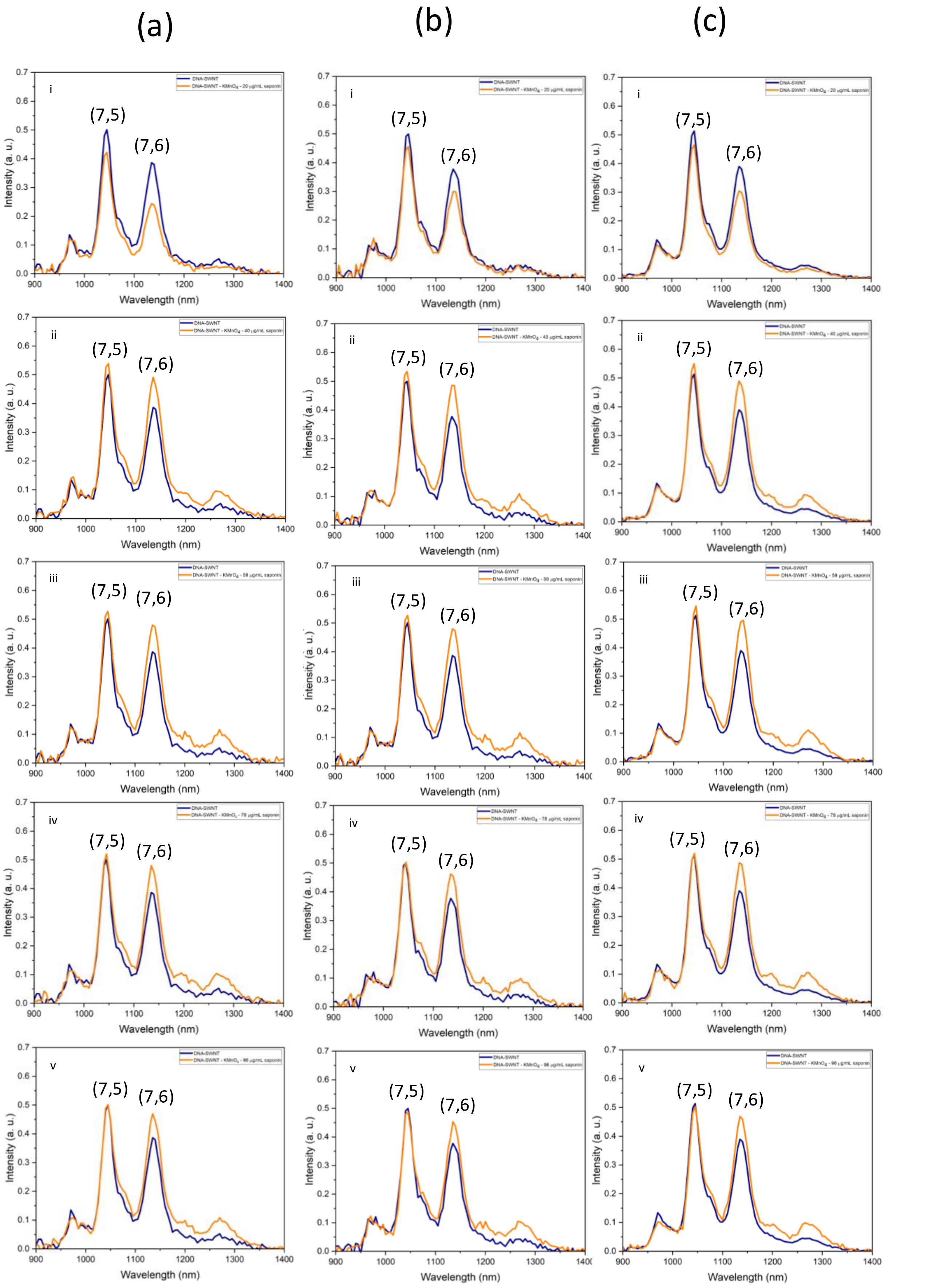

Figure S1 Lin *et al* PL graphs of results of (a) first, (b) second, and (c) third experiments of optical responses of DNA-SWNTs after injecting $KMnO_4$ and saponin solution at the excitation wavelength of 655 nm. Figures (i,ii,iii,iv,v) represent PL graphs of DNA-SWNTs with $KMnO_4$ and saponin solution with final concentrations of 20, 40, 59, 78 and 96 μg/mL respectively.

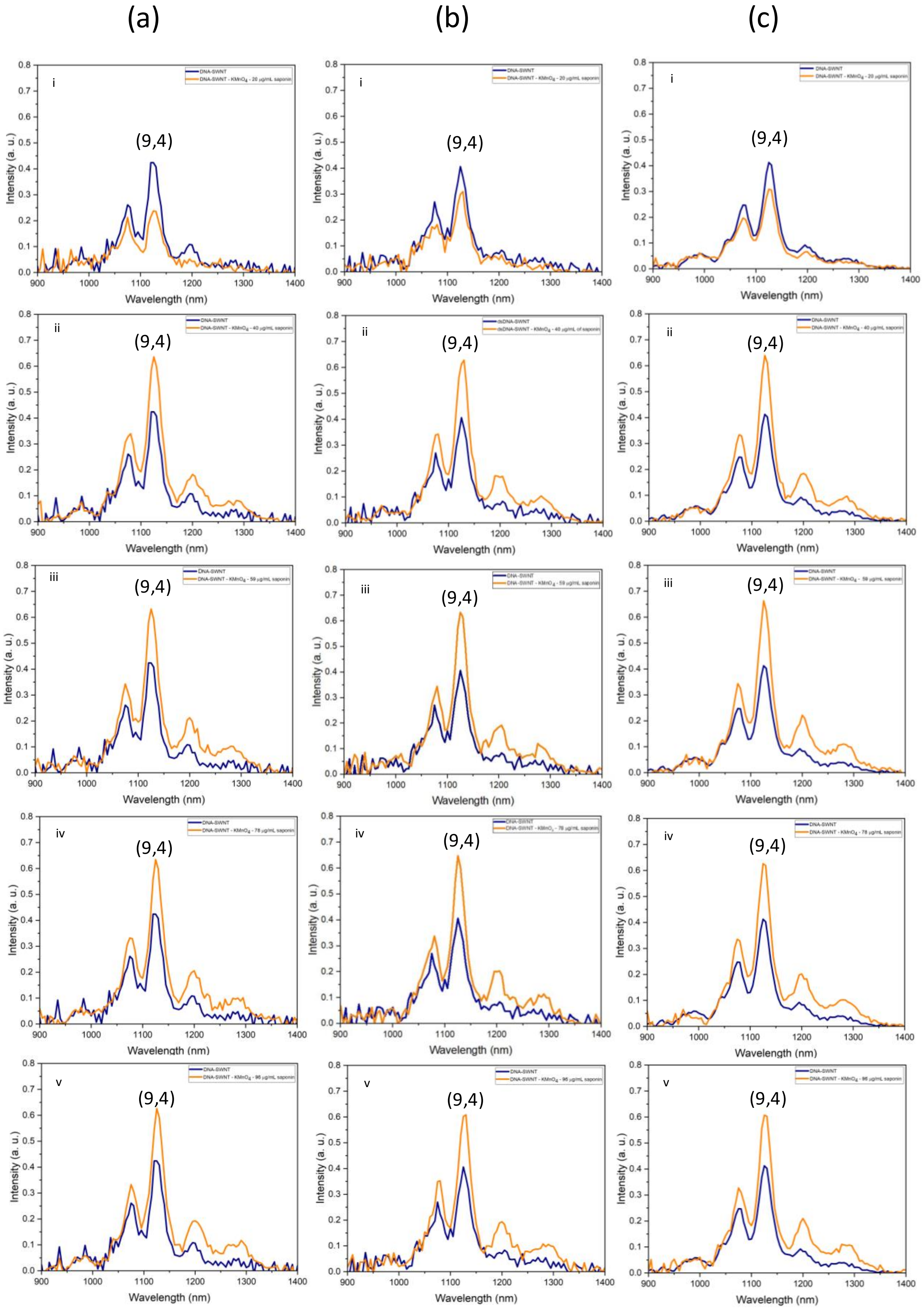

Figure S2 Lin *et al*

PL graphs of results of (a) first, (b) second, and (c) third experiments of optical responses of DNA-SWNTs after injecting $KMnO_4$ and saponin solution at the excitation wavelength of 730 nm. Figures (i,ii,iii,iv,v) represent PL graphs of DNA-SWNTs with $KMnO_4$ and saponin solution with final concentrations of 20, 40, 59, 78 and 96 μg/mL respectively.

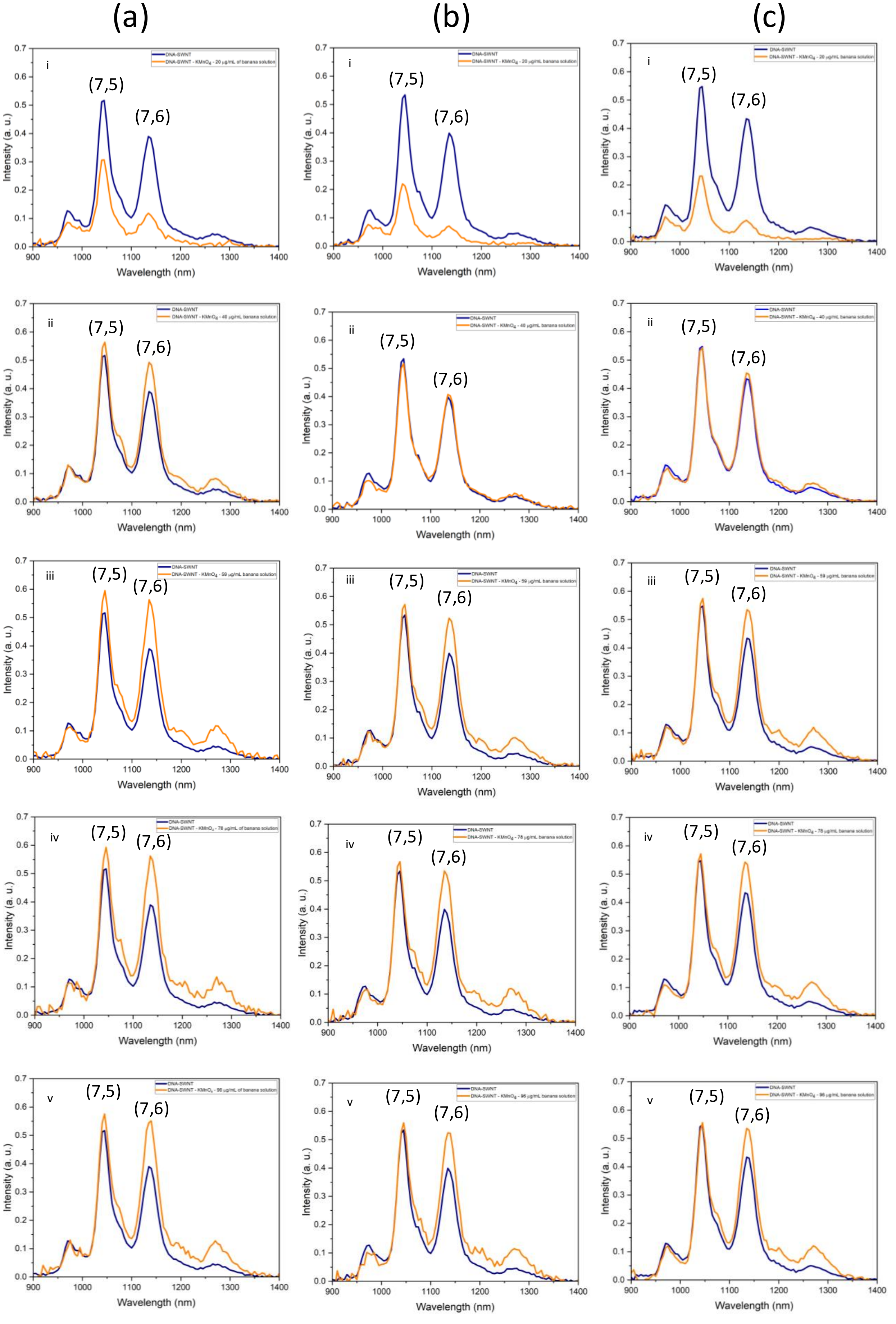

Figure S3 Lin *et al* PL graphs of results of (a) first, (b) second, and (c) third experiments of optical responses of DNA-SWNTs after injecting $KMnO_4$ and banana solution at the excitation wavelength of 655 nm. Figures (i, ii, iii, iv, v) represent PL graphs of DNA-SWNTs with $KMnO_4$ and banana solution with final concentrations of 20, 40, 59, 78 and 96 µg/mL respectively.

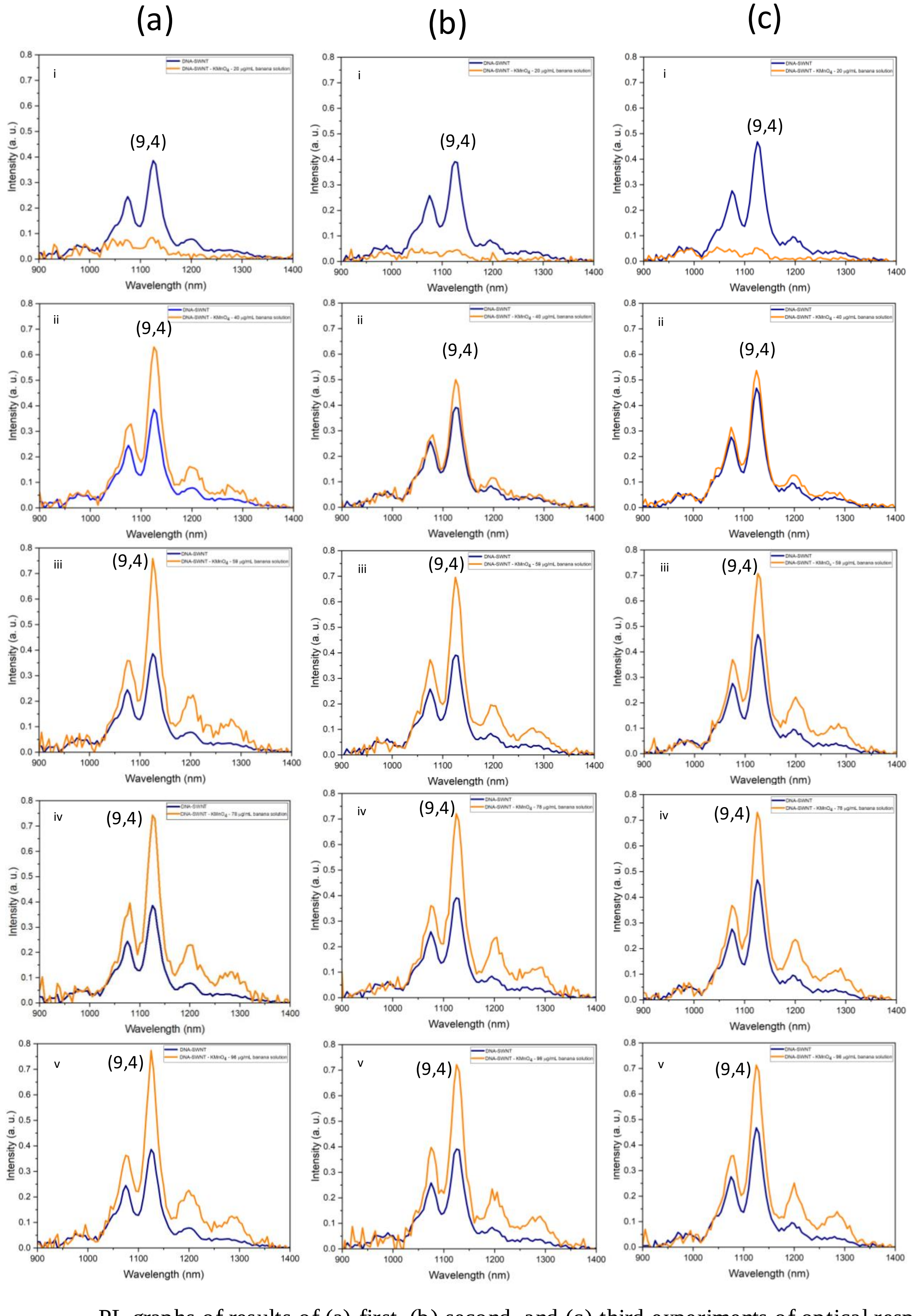

Figure S4 Lin *et al* PL graphs of results of (a) first, (b) second, and (c) third experiments of optical responses of DNA-SWNTs after injecting $KMnO_4$ and banana solution at the excitation wavelength of 730 nm. Figures (i, ii, iii, iv, v) represent PL graphs of DNA-SWNTs with $KMnO_4$ and banana solution with final concentrations of 20, 40, 59, 78 and 96 μg/mL respectively.

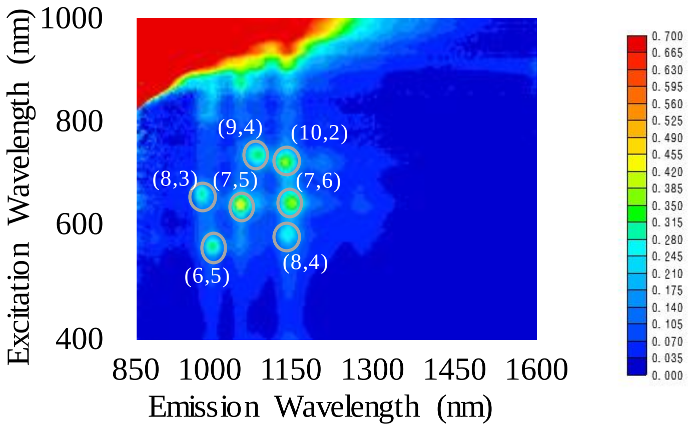

Representation of all remarkable chiralities present in the HiPCO SWNT sample utilized in this research

Figure S5
Lin *et al*

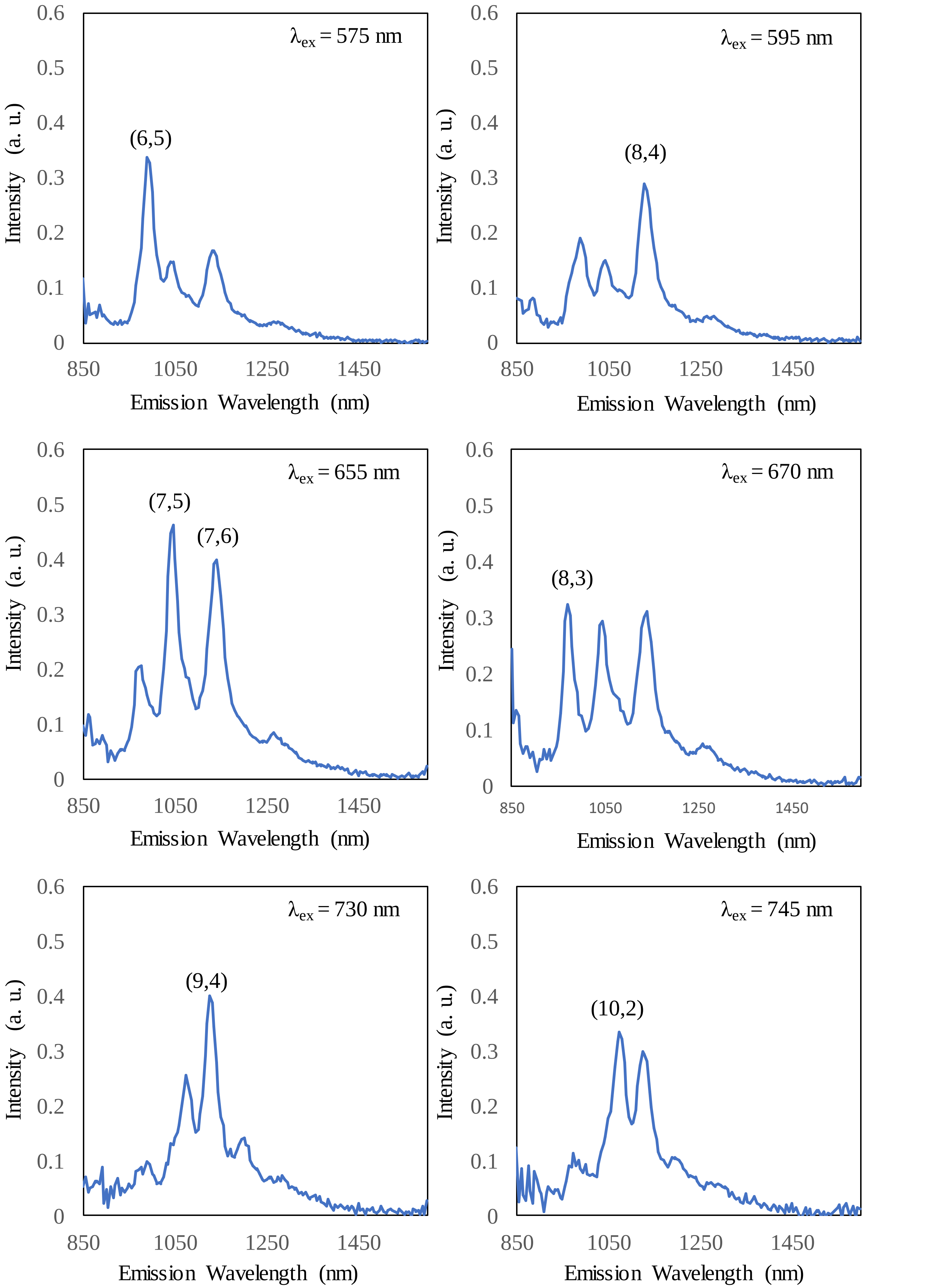

NIR-PL intensity peaks of prominent chiralities of the HiPCO SWNT sample utilized in the research

Figure S6

Lin *et al*

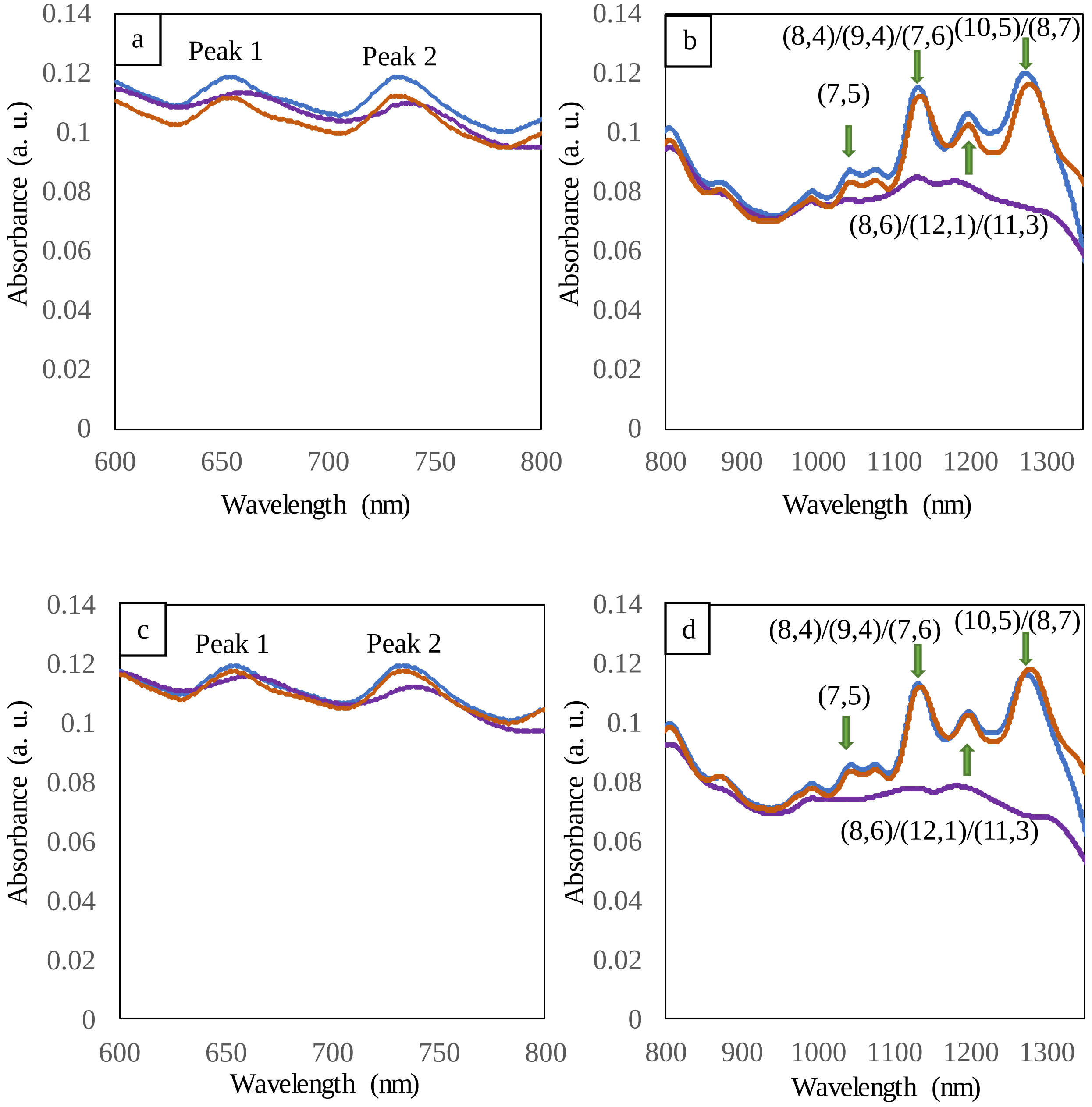

NIR-ABS spectra of DNA-SWNTs after the injection of $KMnO_4$ and (a, b) saponin solution, or (c, d) banana solution. The blue line represents the spectra of DNA-SWNTs (initial state); the purple line represents the spectra after the injection of $KMnO_4$ (final concentration of 0.5 μM); and the orange line represents the spectra after the injection of saponin or banana solutions (final concentration of 59 μg/mL). Figures (a, c) represent E_{22} transitions, whereas figures (b, d) represent E_{11} transitions.

Figure S7
Lin *et al*

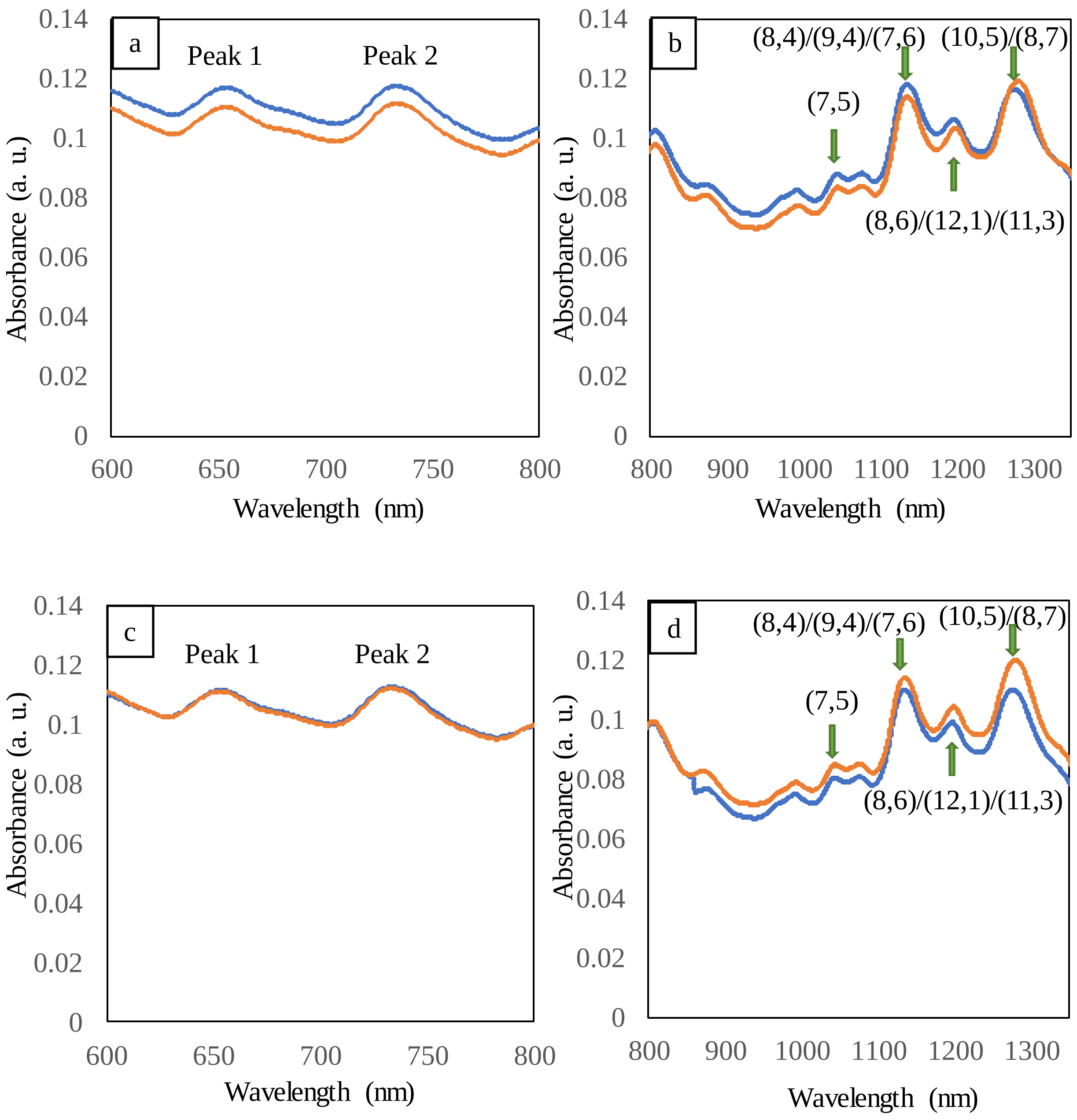

NIR-ABS spectra of DNA-SWNTs after the injection (a, b) saponin solution, or (c, d) banana solution without $KMnO_4$. The blue line represents the spectra of DNA-SWNTs (initial state) and the orange line represents the spectra after the injection of saponin or banana solutions (final concentration of 59 µg/mL). Figures (a, c) represent E_{22} transitions, whereas figures (b, d) represent E_{11} transitions.

Figure S8
Lin *et al*

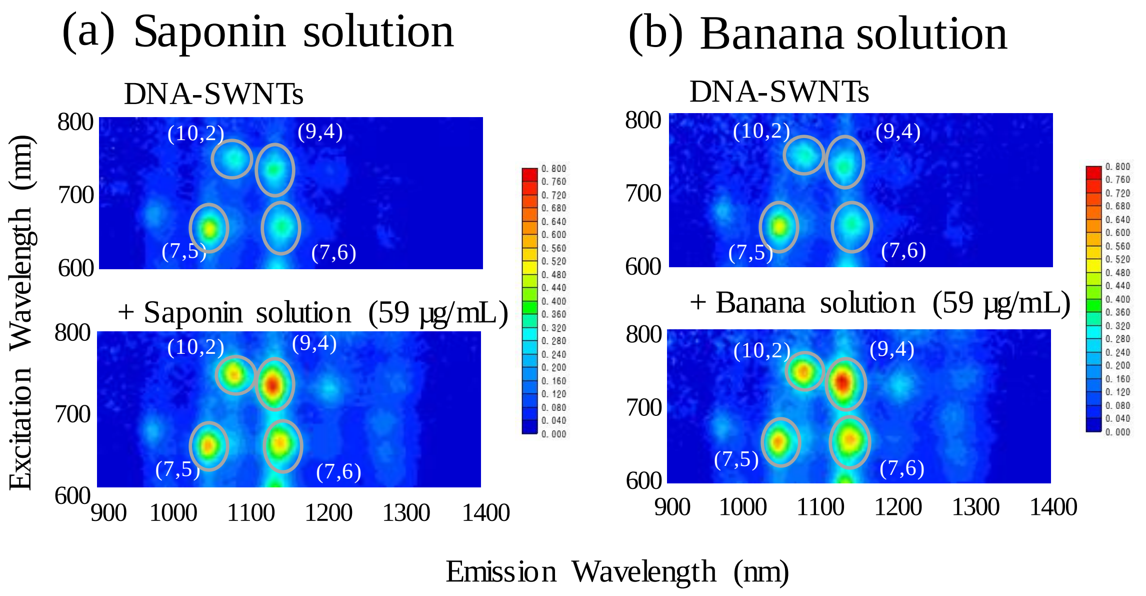

Mapping of the NIR-PL spectra of the DNA-SWNTs with reductants and without oxidant. From the top to the bottom: DNA-SWNTs (initial state), injected solutions of biomolecules (state of reduction). In the state of reduction, the solutions of biomolecules utilized were (a) saponin and (b) banana solutions.

Figure S9
Lin *et al*

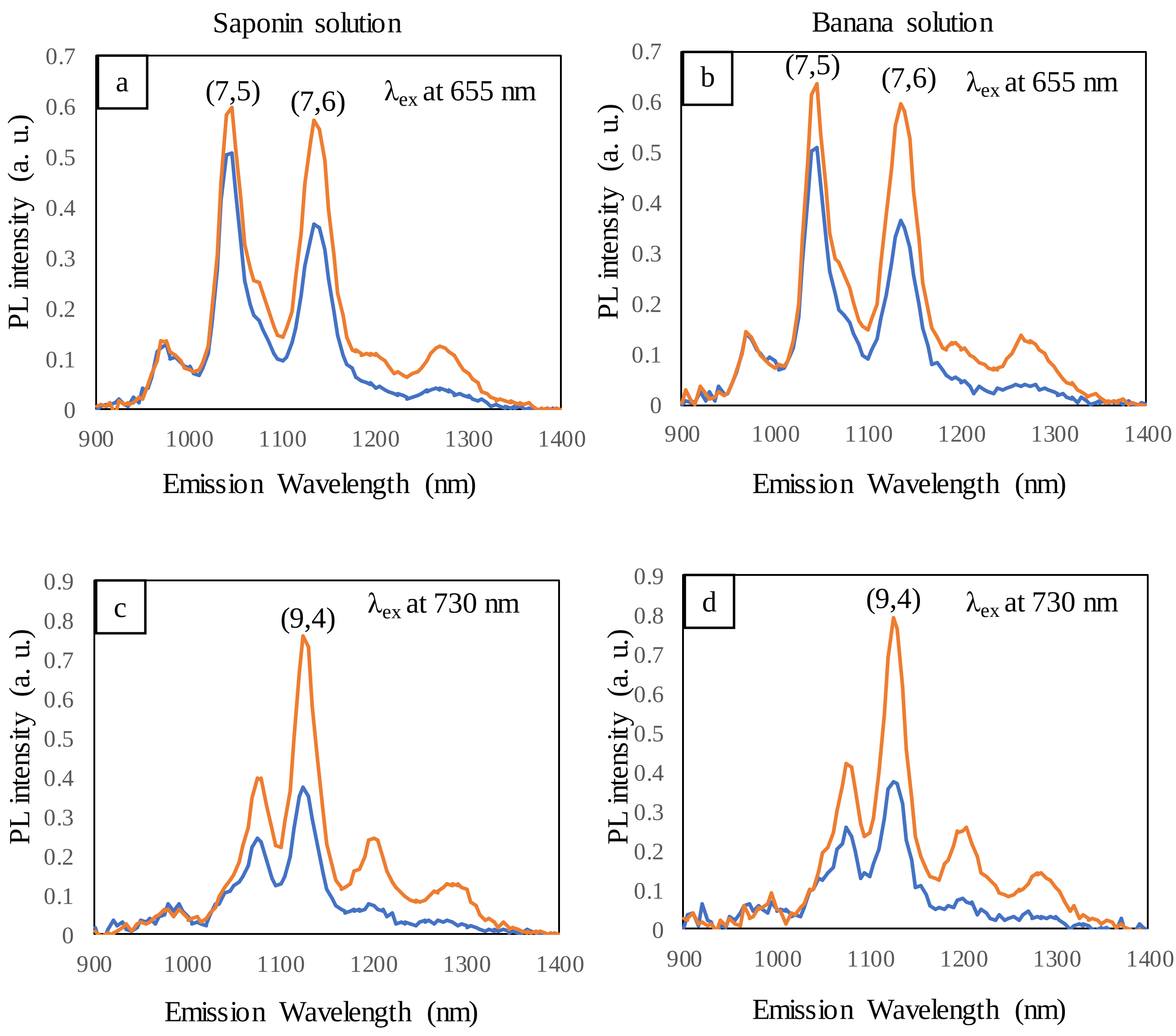

NIR-PL spectra of DNA-SWNTs after the injection of saponin and banana solutions without $KMnO_4$ at the excitation wavelength of (a, b) 655 nm and (c, d) 730 nm. The blue line represents the spectra of DNA-SWNTs (initial state) and the orange line represents the spectra after the injection of saponin and banana solutions (final concentration of 59 μg/mL).

Figure S10
Lin *et al*

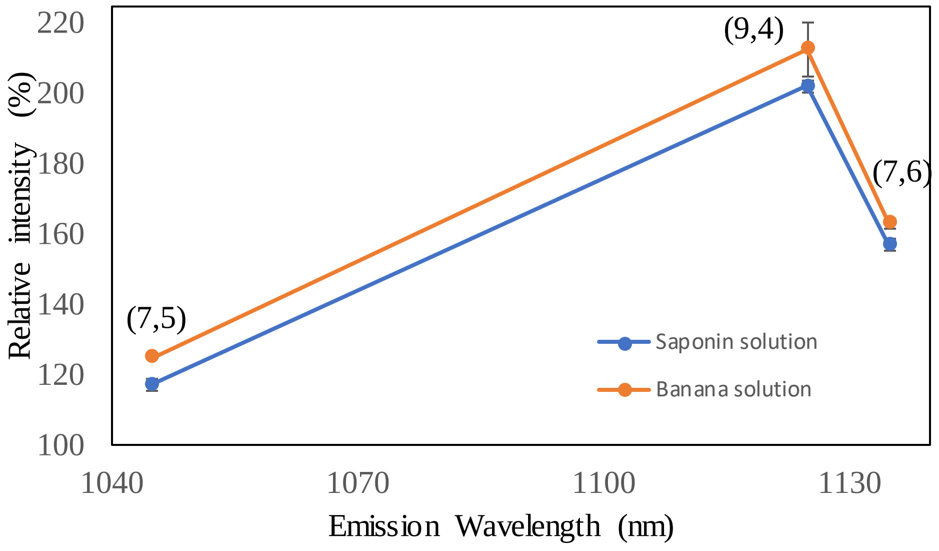

Percentage increases in PL intensities against those of the initial state for (7,5), (7,6) and (9,4) SWNTs at the state of reduction. The reduction processes were carried out without the oxidation process with $KMnO_4$. The final concentration of the solutions of biomolecules was 59 µg/mL each. Error bars were corrected by standard error values.

Figure S11
Lin *et al*

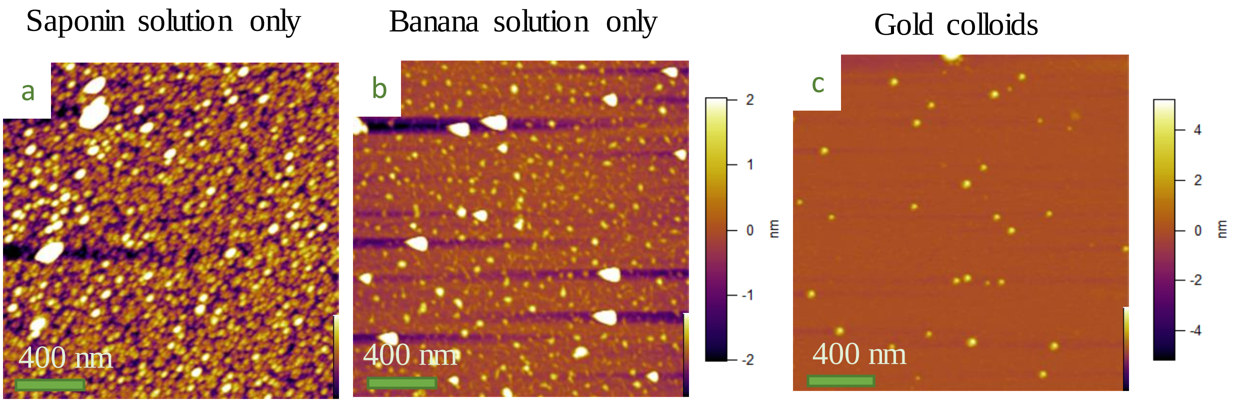

AFM images of (a) saponin solution only, (b) banana solution only, and (c) gold colloids.

Figure S12
Lin *et al*

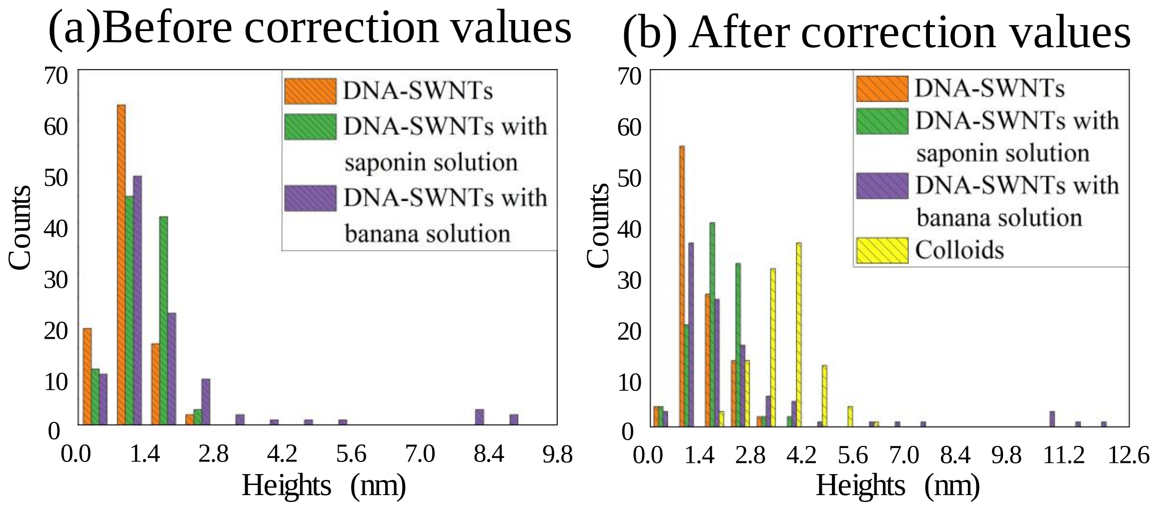

Histograms of DNA-SWNTs before and after mixing with saponin or banana solutions. Histograms were illustrated with the values of (a) the original data without correction in comparison with gold colloids, (b) data corrected in comparison with gold colloids. Each histogram was illustrated with a bin size of 0.7 nm.

Figure S13
Lin *et al*

Table S1: PL intensities and emission wavelengths of DNA-SWNTs before and after addition of $KMnO_4$ and saponin solution

Sample	Excitation Wavelength [655 nm]						Excitation Wavelength [730 nm]		
	(7,5)			(7,6)			(9,4)		
	PL Intensity [a. u.]	%	Emission Wavelength [nm]	PL Intensity [a. u.]	%	Emission Wavelength [nm]	PL Intensity [a. u.]	%	Emission Wavelength [nm]
DNA-SWNTs	0.505 ± 0.008	100	1045	0.384 ± 0.006	100	1135	0.414 ± 0.009	100	1125
DNA-SWNTs w/ $KMnO_4$ and 20 μg/mL saponin solution	0.448 ± 0.022	88.7	1045	0.282 ± 0.034	78.0	1135	0.285 ± 0.042	69.1	1130
DNA-SWNTs w/ $KMnO_4$ and 40 μg/mL saponin solution	0.541 ± 0.008	107.2	1045	0.489 ± 0.003	127.2	1135	0.635 ± 0.006	153.2	1125
DNA-SWNTs w/ $KMnO_4$ and 59 μg/mL saponin solution	0.532 ± 0.013	105.4	1045	0.486 ± 0.009	126.6	1135	0.643 ± 0.018	155.2	1125
DNA-SWNTs w/ $KMnO_4$ and 78 μg/mL saponin solution	0.515 ± 0.010	102.0	1045	0.476 ± 0.013	123.9	1135	0.636 ± 0.011	153.6	1125
DNA-SWNTs w/ $KMnO_4$ and 96 μg/mL saponin solution	0.497 ± 0.010	98.5	1045	0.464 ± 0.009	120.8	1135	0.614 ± 0.011	148.2	1125

Table S1
Lin *et al*

Table S2: PL intensities and emission wavelengths for DNA-SWNTs before and after addition of $KMnO_4$ and banana solution

Sample	Excitation Wavelength [655 nm]						Excitation Wavelength [730 nm]		
	(7,5)			(7,6)			(9,4)		
	PL Intensity [a. u.]	%	Emission Wavelength [nm]	PL Intensity [a. u.]	%	Emission Wavelength [nm]	PL Intensity [a. u.]	%	Emission Wavelength [nm]
DNA-SWNTs	0.533 ± 0.016	100	1045	0.407 ± 0.023	100	1135	0.415 ± 0.046	100	1125
DNA-SWNTs w/ $KMnO_4$ and 20 μg/mL banana solution	0.253 ± 0.047	47.6	1040	0.088 ± 0.026	21.9	1135	0.073 ± 0.018	18.1	1125
DNA-SWNTs w/ $KMnO_4$ and 40 μg/mL banana solution	0.542 ± 0.023	101.8	1045	0.451 ± 0.043	111.1	1135	0.556 ± 0.067	135.4	1125
DNA-SWNTs w/ $KMnO_4$ and 59 μg/mL banana solution	0.581 ± 0.013	109.0	1045	0.540 ± 0.020	133.0	1135	0.721 ± 0.033	175.4	1125
DNA-SWNTs w/ $KMnO_4$ and 78 μg/mL banana solution	0.577 ± 0.014	108.4	1045	0.546 ± 0.014	134.5	1135	0.732 ± 0.011	177.8	1125
DNA-SWNTs w/ $KMnO_4$ and 96 μg/mL banana solution	0.564 ± 0.010	105.9	1045	0.537 ± 0.014	132.1	1135	0.736 ± 0.033	179.2	1125

Table S2
Lin *et al*

Table S3: PL intensities and emission wavelengths for DNA-SWNTs before and after addition of saponin solution

Sample	Excitation Wavelength [655 nm]						Excitation Wavelength [730 nm]		
	(7,5)			(7,6)			(9,4)		
	PL Intensity [a. u.]	%	Emission Wavelength [nm]	PL Intensity [a. u.]	%	Emission Wavelength [nm]	PL Intensity [a. u.]	%	Emission Wavelength [nm]
DNA-SWNTs	0.509 ± 0.009	100	1045	0.366 ± 0.001	100	1135	0.377 ± 0.007	100	1125
DNA-SWNTs w/ 59 μg/mL saponin solution	0.598 ± 0.009	117.3	1045	0.574 ± 0.010	156.9	1135	0.762 ± 0.003	202.0	1125

Table S3
Lin *et al*

Table S4: PL intensities and emission wavelengths for DNA-SWNTs before and after addition of banana solution

Sample	Excitation Wavelength [655 nm]						Excitation Wavelength [730 nm]		
	(7,5)			(7,6)			(9,4)		
	PL Intensity [a. u.]	%	Emission Wavelength [nm]	PL Intensity [a. u.]	%	Emission Wavelength [nm]	PL Intensity [a. u.]	%	Emission Wavelength [nm]
DNA-SWNTs	0.509 ± 0.006	100	1045	0.366 ± 0.003	100	1135	0.375 ± 0.037	100	1125
DNA-SWNTs w/ 59 μg/mL banana solution	0.636 ± 0.004	124.9	1045	0.597 ± 0.005	163.0	1135	0.794 ± 0.030	212.7	1125

Table S4
Lin *et al*

Table S5: NIR absorbances of DNA-SWNTs after the injection of $KMnO_4$ and (a) saponin solution, or (b) banana solution; after the injection of (c) saponin solution, or (d) banana solution without $KMnO_4$ in the wavelength region of 800-1350 nm

Sample	(7,5)			(8,4)/(9,4)/(7,6)			(8,6)/(12,1)/(11,3)			(10,5)/(8,7)		
	Absorbance [a. u.]	%	Wavelength [nm]	Absorbance [a. u.]	%	Wavelength [nm]	Absorbance [a. u.]	%	Wavelength [nm]	Absorbance [a. u.]	%	Wavelength [nm]
DNA-SWNTs	0.0873	100	1043	0.11568	100	1132.5	0.10675	100	1197.5	0.12028	100	1272.5
+ $KMnO_4$ and saponin solution	0.08368	95.9	1043.5	0.11273	97.4	1133.5	0.10279	96.3	1198	0.11648	96.8	1279

(a)

Sample	(7,5)			(8,4)/(9,4)/(7,6)			(8,6)/(12,1)/(11,3)			(10,5)/(8,7)		
	Absorbance [a. u.]	%	Wavelength [nm]	Absorbance [a. u.]	%	Wavelength [nm]	Absorbance [a. u.]	%	Wavelength [nm]	Absorbance [a. u.]	%	Wavelength [nm]
DNA-SWNTs	0.08607	100	1043.5	0.11342	100	1132.5	0.10406	100	1197.5	0.11666	100	1272.5
+ $KMnO_4$ and banana solution	0.08408	97.7	1043.5	0.1125	99.2	1134	0.10309	99.1	1198.5	0.11848	101.6	1279.5

(b)

Sample	(7,5)			(8,4)/(9,4)/(7,6)			(8,6)/(12,1)/(11,3)			(10,5)/(8,7)		
	Absorbance [a. u.]	%	Wavelength [nm]	Absorbance [a. u.]	%	Wavelength [nm]	Absorbance [a. u.]	%	Wavelength [nm]	Absorbance [a. u.]	%	Wavelength [nm]
DNA-SWNTs	0.08826	100	1043.5	0.11846	100	1134.5	0.10673	100	1195.5	0.11688	100	1275.5
+ saponin solution	0.08381	95.0	1043.5	0.11422	96.4	1134.5	0.10382	97.3	1198.5	0.11946	102.2	1280

(c)

Sample	(7,5)			(8,4)/(9,4)/(7,6)			(8,6)/(12,1)/(11,3)			(10,5)/(8,7)		
	Absorbance [a. u.]	%	Wavelength [nm]	Absorbance [a. u.]	%	Wavelength [nm]	Absorbance [a. u.]	%	Wavelength [nm]	Absorbance [a. u.]	%	Wavelength [nm]
DNA-SWNTs	0.08085	100	1043.5	0.11013	100	1134	0.09933	100	1196.5	0.1103	100	1276
+ banana solution	0.08505	105.2	1044	0.11431	103.8	1134	0.10462	105.3	1198.5	0.12018	109.0	1280

(d)

Table S5
Lin *et al*

Table S6: Absorbances of DNA-SWNTs after the injection of $KMnO_4$ and (a) saponin solution, or (b) banana solution; after the injection of (c) saponin solution, or (d) banana solution without $KMnO_4$ in the wavelength region of 600-800 nm

Sample	Peak 1			Peak 2		
	Absorbance [a. u.]	%	Wavelength [nm]	Absorbance [a. u.]	%	Wavelength [nm]
DNA-SWNTs	0.118837	100	654	0.118807	100	733
+ $KMnO_4$ and saponin solution	0.112022	94.3	654	0.112695	94.9	732

(a)

Sample	Peak 1			Peak 2		
	Absorbance [a. u.]	%	Wavelength [nm]	Absorbance [a. u.]	%	Wavelength [nm]
DNA-SWNTs	0.119509	100	654	0.119687	100	733
+ $KMnO_4$ and banana solution	0.117602	98.4	654	0.117762	98.4	733

(b)

Sample	Peak 1			Peak 2		
	Absorbance [a. u.]	%	Wavelength [nm]	Absorbance [a. u.]	%	Wavelength [nm]
DNA-SWNTs	0.117196	100	654	0.117795	100	733
+ saponin solution	0.110764	94.5	653	0.111952	95.0	733

(c)

Sample	Peak 1			Peak 2		
	Absorbance [a. u.]	%	Wavelength [nm]	Absorbance [a. u.]	%	Wavelength [nm]
DNA-SWNTs	0.111839	100	654	0.112806	100	733
+ banana solution	0.11152	99.7	653	0.112357	99.6	733

(d)

Table S6
Lin *et al*
